# Real-World Data for Planning Eligibility Criteria and Enhancing Recruitment: Recommendations from the Clinical Trials Transformation Initiative

**DOI:** 10.1007/s43441-020-00248-7

**Published:** 2021-01-03

**Authors:** Scott R. Evans, Dianne Paraoan, Jane Perlmutter, Sudha R. Raman, John J. Sheehan, Zachary P. Hallinan

**Affiliations:** 1 Biostatistics Center and the Department of Biostatistics and Bioinformatics, Milken Institute School of Public Health, George Washington University, Washington, DC USA; 2 Food and Drug Administration, Silver Spring, MD USA; 3 Gemini Group, Ann Arbor, MI USA; 4 Department of Population Health Sciences, Duke University School of Medicine, Durham, NC USA; 5 Janssen Scientific Affairs, LLC, Titusville, NJ USA; 6 Clinical Trials Transformation Initiative, Durham, NC USA

**Keywords:** Real-world data, CTTI, Recruitment, Eligibility criteria, Trial design, EHR, Claims

## Abstract

The growing availability of real-world data (RWD) creates opportunities for new evidence generation and improved efficiency across the research enterprise. To varying degrees, sponsors now regularly use RWD to make data-driven decisions about trial feasibility, based on assessment of eligibility criteria for planned clinical trials. Increasingly, RWD are being used to support targeted, timely, and personalized outreach to potential trial participants that may improve the efficiency and effectiveness of the recruitment process. This paper highlights recommendations and resources, including specific case studies, developed by the Clinical Trials Transformation Initiative (CTTI) for applying RWD to planning eligibility criteria and recruiting for clinical trials. Developed through a multi-stakeholder, consensus- and evidence-driven process, these actionable tools support researchers in (1) determining whether RWD are fit for purpose with respect to study planning and recruitment, (2) engaging cross-functional teams in the use of RWD for study planning and recruitment, and (3) understanding patient and site needs to develop successful and patient-centric approaches to RWD-supported recruitment. Future considerations for the use of RWD are explored, including ensuring full patient understanding of data use and developing global datasets.

## Introduction

With the cost of developing a single drug estimated between $1.3 and $2.6 billion [1, 2] and more than 80% of clinical trials failing to reach their recruitment targets [3, 4], clinical developers’ interest in the potential of real-world data (RWD) to alleviate drug development challenges is high. Whereas the clinical research enterprise once relied on precedent and other informal sources of information to plan a successful clinical trial, the increasing availability of RWD is creating opportunities for new, more evidence-based ways of planning that have the potential to save time and cost. Using RWD, sponsors and their clinical research organizations can access information such as diagnosis codes, laboratory tests, and histologies to predict the number of patients that could be enrolled and locations where clinical trials could be opened.

Federal incentives for care providers to adopt electronic health records (EHRs), the emergence of several dominant EHR providers, and the consolidation of the US insurance market have expanded the availability of RWD, which now reflect an increasing proportion of the US population. Recent laws, guidance, and regulations, including the 21st Century Cures Act [5], the Prescription Drug User Fee Act VI [6], and the Framework for FDA’s Real-World Evidence Program [7], also support the use of RWD to improve the quality and efficiency of research.

Several characteristics of RWD and studies that use RWD require further critical evaluation. For example, research is needed to comprehensively evaluate whether observational studies using RWD can supplement or replace randomized studies, or whether the focus, quality, and completeness of RWD for measuring outcomes of clinical trial participants results in sufficient study quality. However, there are immediate opportunities for improving the efficiency and potential success rates of traditional clinical trials by informing decisions about eligibility criteria and supporting clinical trial recruitment. These applications of RWD can allow research sponsors to address important questions early in the study design process to help avoid protocol amendments, move away from copy-and-paste eligibility criteria, and ensure the clinical trials enterprise considers eligibility criteria and recruitment strategy upfront within a broader Quality by Design framework [8]. By quantifying eligibility criteria in a real-world population, researchers improve trial feasibility and may increase the generalizability of the results in the process. The US Food and Drug Administration encourages approaches to enhancing inclusiveness so that the clinical trial population more accurately reflects the population that will likely take the drug if it is approved [9].

The remainder of this paper will provide highlights of recommendations and resources developed by the Clinical Trials Transformation Initiative (CTTI) for applying RWD to planning and recruiting for clinical trials, including specific case studies. Some sponsors already routinely use RWD to inform inclusion and exclusion criteria, though there has been little discussion of these efforts in the public domain. Clinical trial recruitment through RWD has rarely been applied to date, producing little documented experience and few examples to model. However, the trends in drug development cost, the scale of EHR and insurance claims data, and recent regulatory guidance combine to make recruitment through RWD an increasingly attractive option.

CTTI is a public–private partnership cofounded by Duke University and the US Food and Drug Administration that seeks to develop and drive adoption of practices that increase the quality and efficiency of clinical trials. In an effort to maximize the opportunities and minimize the challenges associated with the use of RWD to plan eligibility criteria and effectively recruit trial participants, CTTI collaborated with stakeholders across the research enterprise to develop tools to support the use of RWD for these purposes. Activities included qualitative interviews, an expert meeting, and multi-stakeholder project team discussion, following CTTI’s established evidence gathering methodology [10].

Focusing primarily on the use of EHR and insurance claims data for US-based studies of medical products, these recommendations can be used to support in-house selection, organization, and analysis of RWD sets, as well as to improve interactions with data partners and technology providers. The recommendations are also intended to help researchers (1) determine whether RWD are fit for purpose with respect to study planning and recruitment; (2) optimize the use of RWD for study planning and recruitment by engaging cross-functional teams and building out organizational systems and processes; and (3) understand patient and site needs to develop successful and patient-centric approaches to RWD-supported recruitment.

## Overarching Considerations for Using Claims and EHR Data

**Figure 1:**
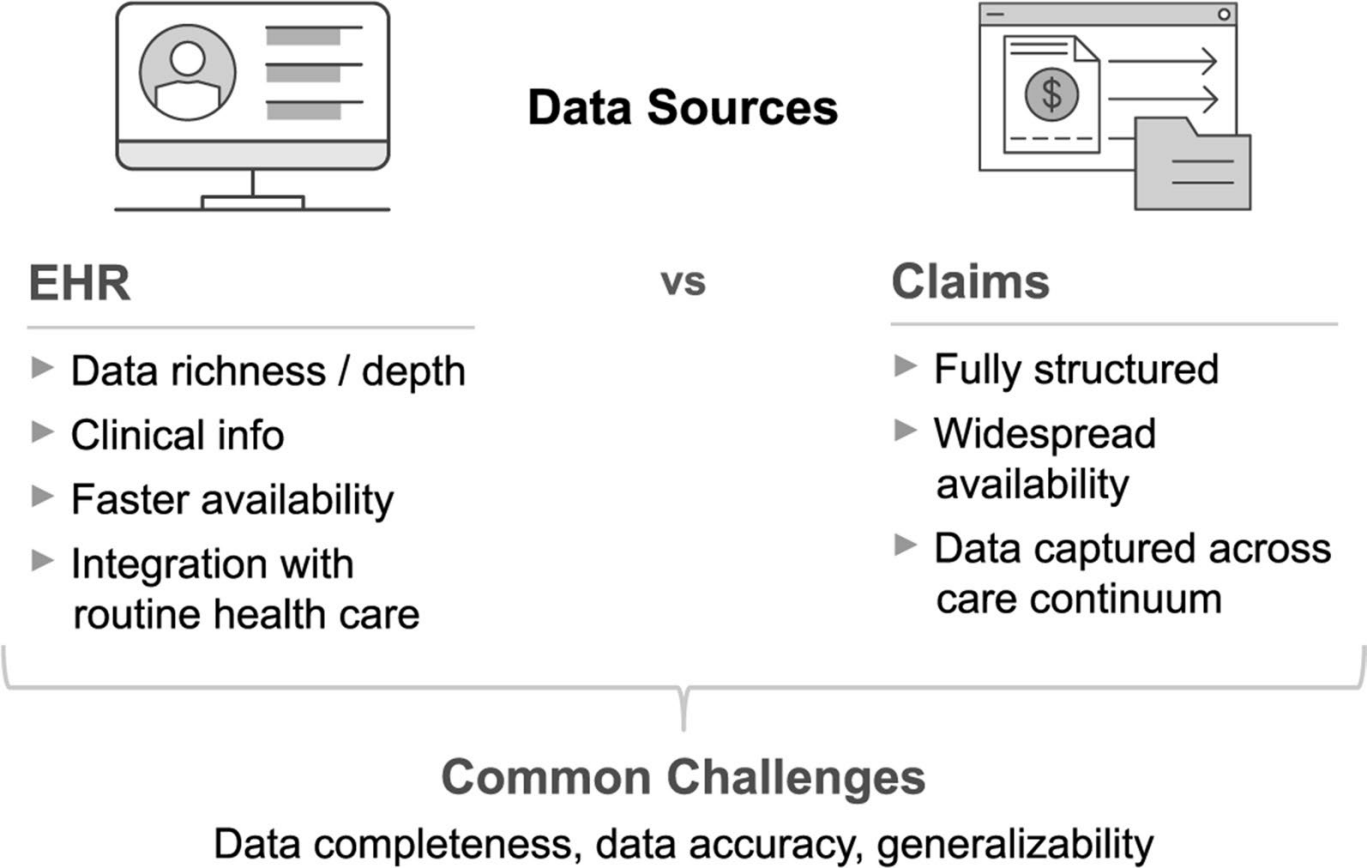
General Characteristics of Data Sources: EHR and Claims

Per the real-world evidence framework released in December 2018 by the US Food and Drug Administration [7], RWD are data relating to patient health status and/or the delivery of health care routinely collected from a variety of sources, such as patient health records and claims. In considering RWD for the planning and conduct of any clinical trial, a baseline understanding of the types of RWD available, as well as the opportunities and limitations of each, is essential. Figure 1 provides an overview of two increasingly available types of RWD, which are the focus of the current work: EHR and claims data. Although the specific advantages and disadvantages of EHR and claims data will vary from one data source to another, the figure presents a useful starting point for understanding the general characteristics of each.

This work offers considerations for using RWD in determining eligibility criteria and recruitment strategies; however, these data bring limitations. EHRs are often unstructured and can be challenging to work with, as data may need to be aggregated across multiple providers or disparate sources (e.g., radiographic images, genomic data, laboratory results). Data completeness and accuracy can vary for a given patient (e.g., if they see several providers), and across provider organizations. While fully structured, claims are limited in depth and richness of data due to their primary utility being billing. Lapses in coverage risk these data being inaccurate or incomplete, and claims are subject to a time lag (30 to 90 days or more) to allow for adjudication. Finally, with all types of RWD, the suitability of data is only one of the necessary considerations. For example, the information gathered by talking to people, and particularly patients, principal investigators, and study coordinators, is also essential. These insights offer understanding of the where and how the data are used, the disease burden and care trajectories of patients and what may or may not be feasible for a given effort. While the limitations addressed here should not be deterrence from using RWD for planning eligibility and recruitment, they should be recognized.

## General Principles for Using RWD

Ideally, research sponsors should begin seeking insights from RWD early in the product lifecycle (e.g., prior to initiating phase 1 trials or feasibility studies) to characterize relevant patient populations and subpopulations and understand unmet need. For many teams, this is a departure from the standard way of working and may be seen as unnecessary and time-consuming. However, there is a benefit in that resulting insights can help facilitate data-driven decisions at the study level in a way that does not delay start-up timelines, while also allowing study teams to identify potential pitfalls or false assumptions that can cause delays in later phases.

When considering potential opportunities to leverage RWD to support collaborative study designs, it is critical to engage a cross-functional team, ideally including clinical, operations, epidemiology, biostatistics, informatics, and data science perspectives, as well as external perspectives from patients and sites [11]. A team with diverse expertise will ensure insights gleaned from RWD are considered holistically, through a variety of lenses, and appropriately challenged when necessary prior to team action. A cross-functional team that is established to interpret RWD, extract insights, and plan recruitment strategies will allow sponsors to apply the data in a way that supports the best path forward from the outset. For example, in one case study explored by CTTI [12], an industry research sponsor used its in-house expertise to determine that EHR data, not claims data, presented the best solution to determine whether to expand the study eligibility criteria for a clinical trial it was conducting. Challenged with a slow recruiting trial exploring the safety of breast cancer immunotherapy combinations, the team needed to quickly determine if it was unnecessarily excluding patients. The sponsor’s protocol required eligible patients be treated with a hormone therapy in second-line, which was a popular treatment option at the time the study was designed. However, since that decision, a CDK4/6 inhibitor had come onto the market. The team suspected this new medication’s use in combination with the hormone therapy as first line treatment was the criterion making patients ineligible. Given the breast cancer treatment in question was only recently introduced to the market, the study team needed data that were both recent (ideally within the past 2 months) and quickly available. EHRs were not only timely, but also available in-house to the sponsor with biomarker testing algorithms in place to quickly define breast cancer sub-types. While claims data were also available to the sponsor, those would not show a patient’s hormone receptor status, so the team would have to use treatment as a proxy for that information. Using the EHRs, the study team plotted the data showing trend lines over each year. The data showed that there was a substantial increase in the use of the newly marketed therapy, and its use was impacting eligibility. This drove an internal discussion around the risks and benefits of including patients on a third-line therapy, knowing that this addition would merit a protocol amendment. The team ultimately decided that although the amendment would be costly, it was an investment that was likely to pay off. Once the trial concluded, the team reported that the expansion of eligibility criteria to add the third-line treatment likely boosted recruitment rates.

## Using RWD to Plan Eligibility Criteria

Sponsors considering RWD as a resource to plan eligibility criteria should carefully evaluate available data against the needs of the particular study. Appropriate questions to consider in this stage include (1) identification of eligibility criteria available in RWD; (2) how the recency and generalizability of available data impact the study in question; and (3) the potential for use of proxy measures to understand variables of interest not typically captured in EHRs and claims data.

As demonstrated in another case study explored by CTTI [13], analysis of relevant RWD early in the study design stage can help to avoid common pitfalls. In this global, phase 2 clinical trial evaluating a biological treatment for an inflammation indication, the study team analyzed RWD to assess their planned eligibility criteria. They had assumptions about how to define the patient population based mostly on anecdotal evidence and previous experience. Those assumptions drove the choice of a particular age criterion, and the team decided to use vendor-sourced EHR data early on in the protocol design phase to understand the clinical attributes and profile of the eligible cohort. The EHRs came predominately from the United States, which proved to be a sufficient starting point to guide decision-making for the global study. The team then utilized patient-level claims data for a deeper, more targeted analysis and assessment of patient characteristics and developed an interactive visualization that enabled the team to assess the impact of different criteria. One question of interest was how including or excluding concomitant or prior treatments would impact the size of the eligible patient population. The result of the study team’s analysis was that the strict age criterion would exclude one-third of their potential participant population. After confirming from experts in the therapeutic area that there were no concerns about including older participants, they did—and likely saved significant time and cost while generating a more representative study population.

In another case study for a global, phase 3 endocrinology trial [14], the sponsor used RWD to analyze anticipated access to patient populations across countries in Asia, Europe, and North America. Starting early in the draft protocol stage (with the primary endpoint fully specified, but secondary and exploratory endpoints still flexible), the study team analyzed data from two distinct federated EHR systems: one with a combination of United States and European data and the other with European data only. They also examined historical studies and competitor trials using publicly available sources. The goal was to target the most representative populations in the least restrictive manner that still achieved the study’s endpoints. A comparison of screen failure rates of historical studies alongside the EHRs confirmed that one eligibility criterion, related to use of a particular background medication, would result in exclusion of a significant proportion of patients from the trial. The team brought these findings to investigators, sites, and patients to determine whether they aligned with these stakeholders’ experiences and if there were any important insights into the burden of procedures or other requirements that should be factored into eligibility considerations. The resulting decision was that the study team could not adjust the problematic eligibility criterion because it was central to the study’s aims. However, the study team alerted the investigative sites, allowing them to fully prepare for associated recruitment challenges.

## Using RWD to Support Recruitment

Though limited, evidence suggests that RWD-supported recruitment strategies—such as direct email campaigns to patients identified through claims data and EHR-supported discussions at the point of care—have the potential to increase recruitment effectiveness and efficiency for many trials [15, 16]. CTTI recommends incorporating RWD-supported recruitment, alongside traditional modes of recruitment, whenever available data are fit for purpose. In determining fitness, the context of the proposed use of the RWD should guide the threshold for decision-making. By and large, the same considerations that make RWD fit for purpose with respect to evaluating eligibility criteria also apply when selecting RWD to optimize recruitment. There are, however, additional considerations when data are used for recruitment purposes. Most importantly, there must be an appropriate pathway to contact potential participants while maintaining privacy.

**Figure 2:**
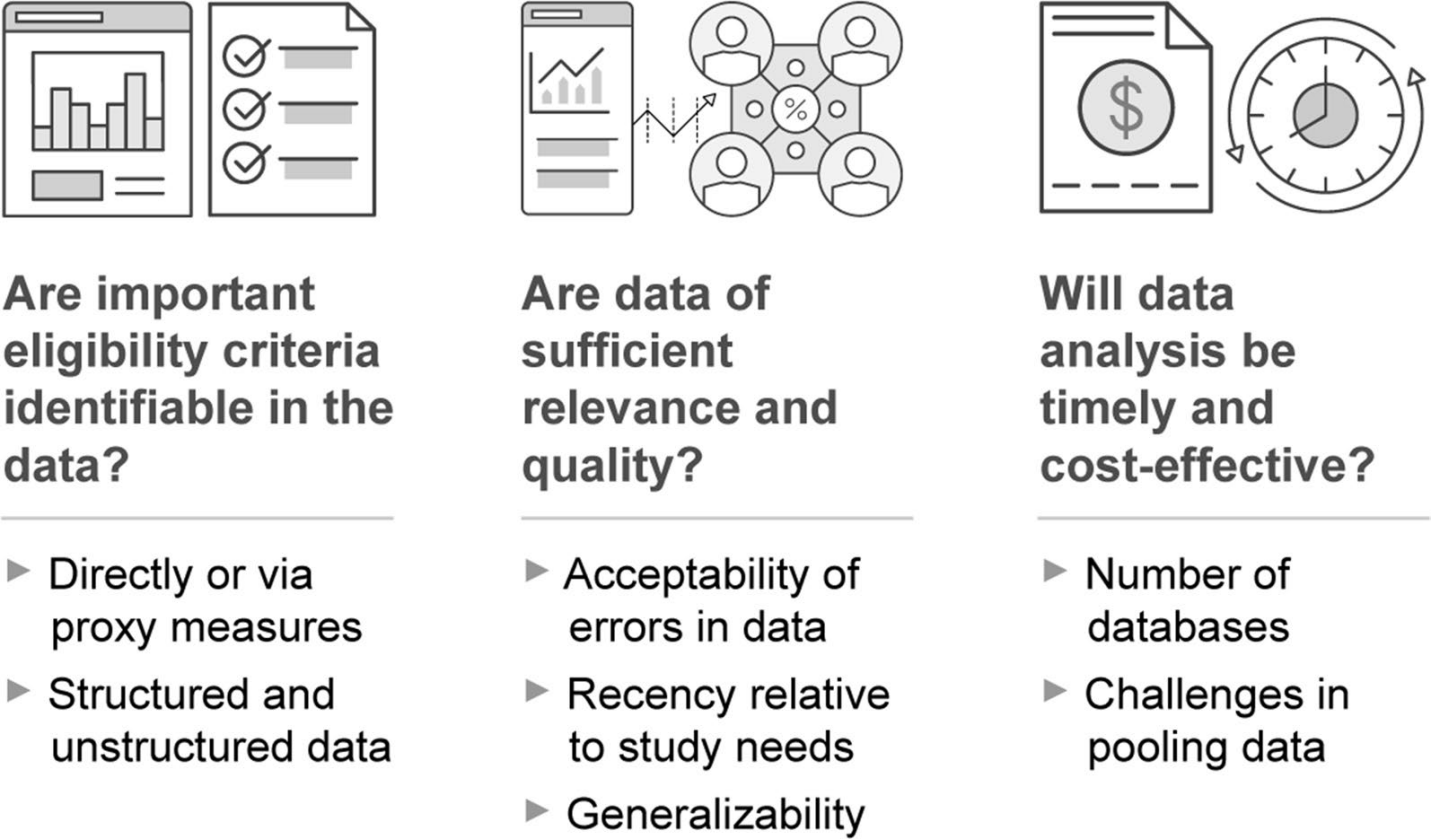
Fit-for-Purpose Data

Sponsors should especially consider using RWD-supported recruitment strategies for any trial likely to face recruitment challenges (e.g., small target population, high screen failure rates anticipated, short timelines for meeting enrollment targets). Implementation of these strategies, which we will explore in detail momentarily, can be particularly beneficial in challenging scenarios. To reap these benefits, it is still necessary to assess what eligibility criteria are necessary and feasible, as addressed above. Low or slow recruitment that stems from unrealistic eligibility criteria will still be a problem, even with RWD-supported recruitment. To assist in determining whether data will be fit for purpose, CTTI offers a tool highlighting relevant considerations (Fig. 2).

In assessing whether RWD sources are fit for purpose, sponsors should remember that exactly matching patient information in EHR or claims data to trial inclusion and exclusion criteria will often prove impossible, so using RWD to identify eligible patients requires tolerance for a certain degree of error. Sponsors can address false positives in most cases with systems to confirm eligibility of identified patients prior to enrollment (e.g., screening calls with study coordinators).

A final consideration: RWD offer unique opportunities for both massive scale (e.g., contacting tens of thousands of patients through claims databases) and highly individualized and timely communication (e.g., facilitated through the EHR system during routine medical appointments). In planning RWD-based recruitment strategies, sponsors should engage with the full range of stakeholders, from patients to institutions and institutional review boards (IRBs), to understand what approaches will work best, while still respecting patient privacy and perception of recruitment messaging. Selecting appropriate communication channels should be done in close consultation with patients, caregivers and other stakeholders—including IRBs and institutions—during the study planning process. RWD-related questions to discuss with patients may include: “What level of personal interaction is needed to feel comfortable enrolling?” “Will the communication be perceived as an invasion of privacy?” and “What expectation does the communication approach set for the patient?” For example, in appropriate circumstances, low-touch recruitment approaches may allow large numbers of potential participants to be recruited in relatively short periods of time and at relatively low cost per patient; however, these approaches may also make retention more challenging—participants may be less likely to stay involved in a study if they have invested less upfront. Early engagement with patients during study planning can help to clarify tradeoffs and plan accordingly.

**Figure 3:**
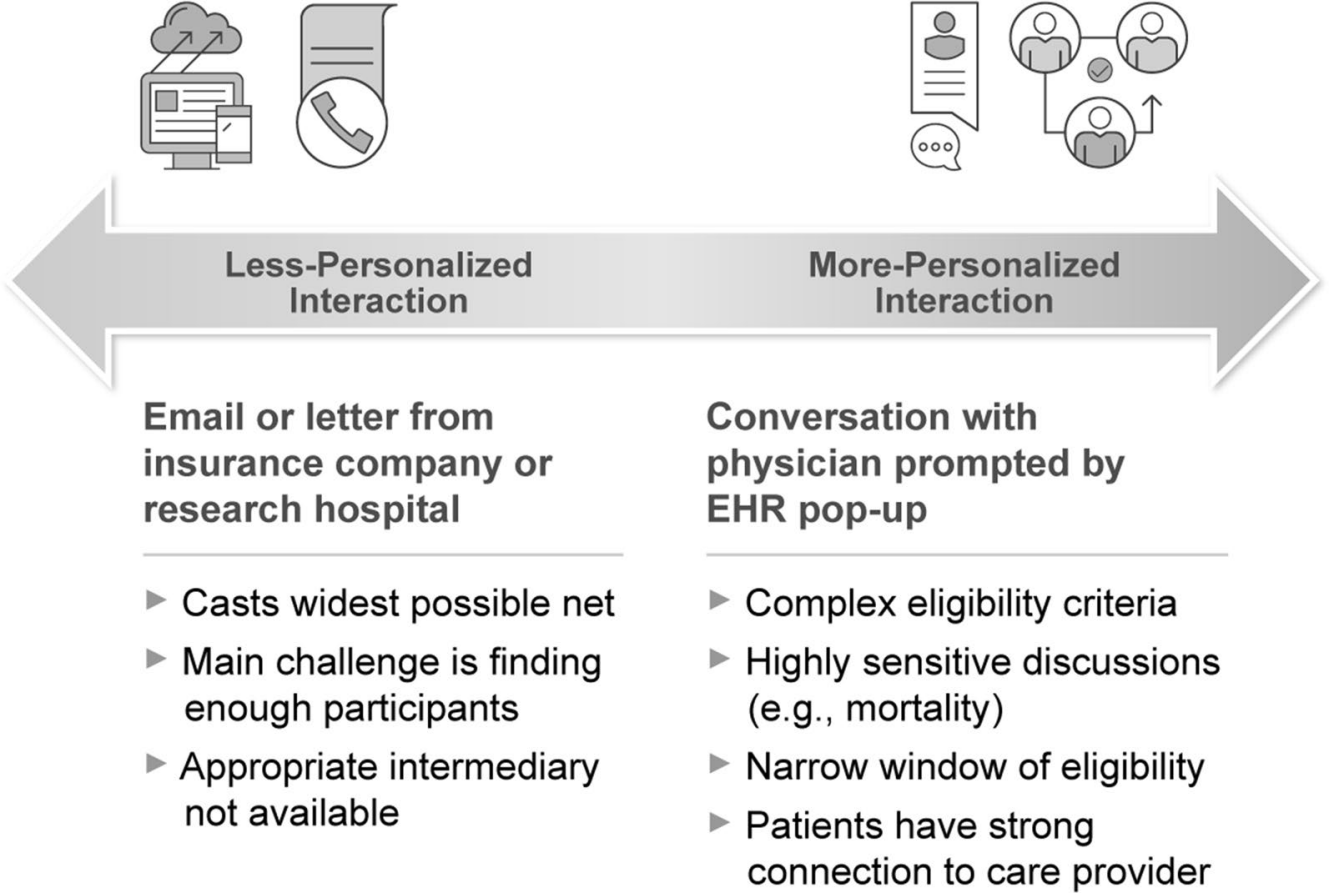
Planning RWD-Supported Recruitment

Datasets can be effectively used for recruitment only if there is an appropriate pathway to contact patients; and for the subset of RWD where this is possible, successful use often requires partnership with the dataset owner (e.g., local investigators and research staff, health system, insurance company) and an understanding of privacy regulations. To be usable for recruitment purposes, data sources must also provide an appropriate pathway to re-identify patients while protecting health information. Early conversations with IRBs or documentation of previously approved trials that used similar recruitment approaches are strongly advised. In circumstances where researchers are working with anonymized or de-identified data to build a cohort of patients meeting trial entry criteria, re-identification and contacting individual patients should be completed by a point of contact appropriate to the data source (e.g., providers might contact patients identified through EHRs, and payers might contact patients identified through claims data). While RWD-based strategies bring unique opportunities to automate and precisely target recruitment efforts (Fig. 3), and should be planned accordingly, they should also still follow standard recruitment best practices [17].

## Looking Ahead: Enhancing RWD Capabilities for the Research Enterprise

The utility of RWD will increase as data linkage expands, allowing researchers to build a more complete picture of the landscape of patient health. Simultaneously, the technology to bring more automation into the RWD-capture process will soon help make RWD scalable. Yet with these advances also come challenges, such as transparency. For example, the patient community may not generally be aware of how their data are being used. To facilitate greater understanding and acceptance by patients of the use of RWD for a variety of research-related purposes, stakeholders should work together to identify best practices that ensure patients are aware of how their data are shared and, when possible, allow patients to make informed decisions regarding use of their data.

As a long-term goal, the identification and support for approaches to create patient-protected global datasets, though challenging, would bring tremendous value to research and public health.

## Conclusion

RWD provide an important tool for the research community to improve the planning and conduct of clinical trials, and Table 1 summarizes recommendations for use. The expanded availability of RWD sources and encouragement by regulators suggest that using RWD to optimize eligibility criteria and recruitment may become the “new normal” across the clinical trials enterprise. The stepwise approach outlined in this paper provides sponsors with the opportunity to develop new capabilities and expand existing ones. The benefits include more efficient clinical trials and the potential to expand clinical trial access by broadening eligibility criteria, reducing patient and site burden, and speeding up the development and availability of new medical treatments.

**Table 1:** Summary of Recommendations

Principles for Using RWD	Using RWD to Plan Feasible Eligibility Criteria	Using RWD to Support Recruitment
1. Begin seeking insights from RWD as early as possible 2. Use RWD to complement and support collaborative study design	1. Evaluate available RWD sources against the particular needs of the study being planned 2. Use RWD to identify and test important assumptions about the impact of potential eligibility criteria on trial feasibility 3. Plan for iterative, targeted team discussions starting early in protocol design	1. Start by designing realistic eligibility criteria 2. Incorporate RWD-supported recruitment strategies whenever feasible 3. Understand and address the needs of patients and sites with respect to RWD-supported recruitment
Enhancing RWD Capabilities for the Research Enterprise		
1. Identify opportunities and risks of enhanced data linkage 2. Support continued development of underlying technology 3. Evaluate RWD-supported recruitment strategies and identify best practices 4. Explore transparency of secondary data use to the patient community and opportunities to enhance patient agency with respect to usage of their data 5. Enhance communication channels for RWD-supported recruitment 6. Identify opportunities to increase diversity of study participants 7. Identify and support approaches for creating global datasets		

## Abbreviations

RWD: Real-world data
CTTI: Clinical Trials Transformation Initiative
EHR: Electronic health records
